# Transverse colon volvulus, radiological diagnosis, and surgical management: a case report

**DOI:** 10.1093/jscr/rjaf918

**Published:** 2025-11-26

**Authors:** Suhair Isam Abdalla Mohamed, Masud Awil, Hussein Ahmed

**Affiliations:** Department of Radiology, Newham Hospital, Barts NHS Trust, Glen Rd, E13 0SQ, London, United Kingdom; Department of Radiology, Newham Hospital, Barts NHS Trust, Glen Rd, E13 0SQ, London, United Kingdom; Department of General Surgery, Newham Hospital, Barts NHS Trust, Glen Rd, E13 0SQ, London, United Kingdom

**Keywords:** transverse colon volvulus, bowel obstruction, whirl sign, CT scan, colectomy

## Abstract

Transverse colon and splenic flexure volvuli are rare causes of bowel obstruction. Diagnosing transverse colon volvulus is often challenging because it is uncommon and presents with unusual clinical signs. Usually, clinicians make the final diagnosis during surgery. Delays in diagnosis significantly increase the risk of complications and death. Imaging, particularly computed tomography scans, plays a vital role in diagnosis, as it provides detailed anatomical information, identifies characteristic features, and facilitates early intervention, which is essential in preventing complications and improving patient outcomes. We present a case of a 79-year-old woman who arrived with acute abdominal pain. Prompt imaging allowed us to perform surgery in a timely manner. This case underscores the importance of radiological suspicion in diagnosing rare causes of bowel obstruction.

## Introduction

Transverse colon and splenic flexure volvuli are rare causes of bowel obstruction [1]. Diagnosing transverse colon volvulus (TCV) is challenging due to its rarity and the unusual clinical presentation, with a definitive diagnosis typically made during surgery. Delays in diagnosis significantly increase the risk of complications and death [2]. Imaging, especially computed tomography (CT) scans, plays a crucial role in diagnosis [3]. This case underscores the importance of radiological suspicion in identifying uncommon causes of bowel obstruction.

## Case report

A 79-year-old woman presented with a 4-day history of suprapubic abdominal pain and vomiting. During this period, she had not passed stool and reported only minimal flatus. There were no associated fevers, chills, or urinary symptoms.

Her past medical history included neurofibromatosis type I, epilepsy, and a previous appendectomy.

On examination, the abdomen was soft with mild tenderness in the right upper quadrant and epigastrium. There was no abdominal distension or evidence of peritonitis. Rectal examination revealed solid stool without blood.

Initial laboratory investigations showed: hemoglobin 151 g/l, white cell count 14 × 10^9^/l, platelets 339 × 10^9^/l, international normalized ratio (INR) 1.1, sodium 141 mmol/l, potassium 3.9 mmol/l, and C -reactive protein (CRP) 10 mg/l.

**Figure 1:**
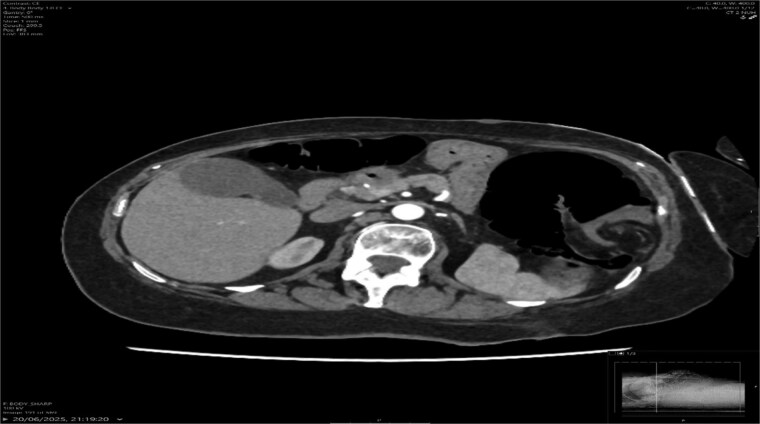
Axial CT showing the swirl sign.

**Figure 2:**
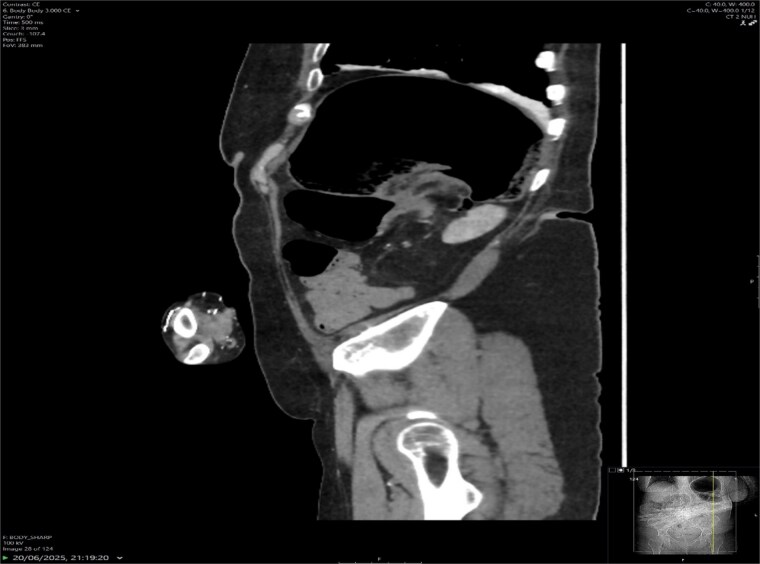
Sagittal CT showing swirl sign and dilated large bowel loops.

**Figure 3:**
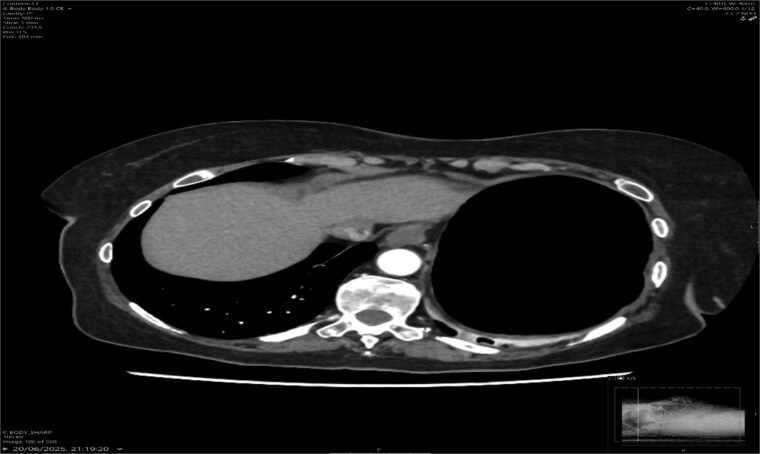
Axial CT showing dilated transverse colon at the splenic flexure.

**Figure 4:**
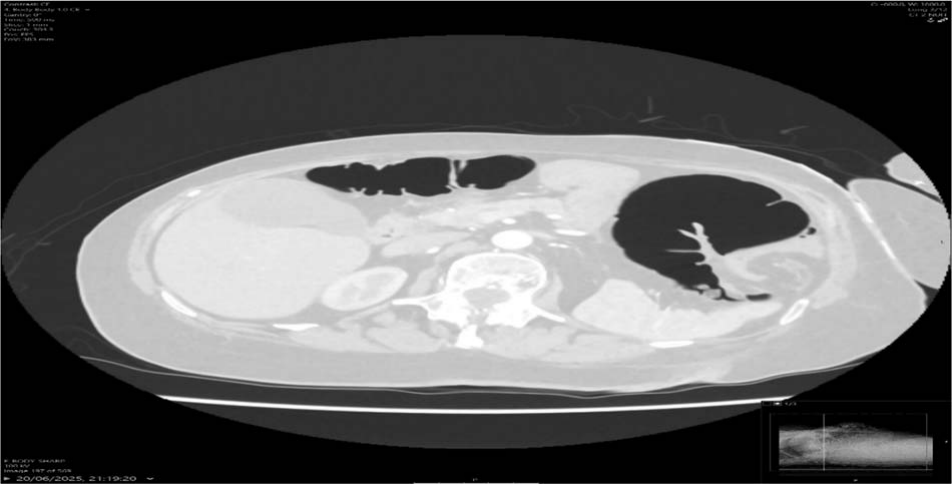
Axial CT lung window showing no evidence of perforation.

A contrast-enhanced CT scan of the abdomen demonstrated a whirl sign at the mesenteric root (Figs 1 and 2), dilated transverse colon with a transition point at the splenic flexure (Fig. 3), and no evidence of ischemia or perforation (Fig. 4). These findings were consistent with TCV.

The patient underwent an emergency laparotomy with subtotal colectomy. At laparotomy, a volvulus of the transverse colon was identified, trapped between the spleen and left hemidiaphragm. The colon was grossly distended with ischemic changes extending into the splenic flexure. The sigmoid was redundant with an elongated mesentery, predisposing to future volvulus. Multiple congenital adhesions were seen between small and large bowel loops. Neurofibromatous lesions were observed on the serosal surface of the stomach and small bowel, consistent with the patient’s background of neurofibromatosis type I.

Given the extensive distension, ischemia, and high likelihood of recurrence with segmental resection, the surgical team opted for a subtotal colectomy with ileosigmoid anastomosis rather than a limited segmental resection. This approach aimed to remove redundant colon, minimize recurrence risk, and address ischemic areas comprehensively.

Postoperatively, the patient initially recovered but subsequently developed an anastomotic leak, necessitating re-laparotomy. Findings included contamination with small bowel adhesions and perforation near the anastomosis. The anastomosis was dismantled, the distal colon closed, and an end ileostomy was fashioned. She was managed in the ICU with antibiotics, IV fluids, and supportive care. Her recovery thereafter was gradual, with stabilization following the second surgery.

### Histopathology findings

Histopathology of the subtotal colectomy specimen demonstrated widespread ischemic necrosis involving both small and large bowel. Sections from the anastomotic site revealed ischemia involving the full thickness of the bowel wall, serositis, and inflammatory exudate, with associated fibrosis of the attached mesentery. Reactive lymph nodes were identified, but there was no evidence of dysplasia or malignancy. A benign submucosal lipoma was also noted.

Importantly, serosal neurofibromatous lesions were confirmed, consistent with the intraoperative findings. These lesions have rarely been reported in association with volvulus and may have contributed to altered bowel motility and adhesions. 

## Discussion

TCV is an extremely rare cause of large bowel obstruction, with fewer than 100 cases reported in the literature [2, 4]. Because of its rarity, diagnosis is often delayed, and mortality may reach 30%–33% in complicated or late cases [2]. The condition is predisposed by colonic redundancy, chronic constipation, adhesions, neurological disorders, and congenital anomalies [5].

In our patient, the presence of congenital adhesions, redundant sigmoid mesentery, and neurofibromatosis lesions likely contributed to volvulus formation. Neurofibromatosis type I (NF1) has well-established gastrointestinal manifestations in up to 25% of patients, including neurofibromas, gastrointestinal stromal tumors, vascular lesions, and adhesive disease [6]. Although gastrointestinal involvement is relatively common in NF1, its direct association with volvulus is rare. Only isolated reports exist, such as recurrent sigmoid volvulus in NF1 [7] and ileal volvulus secondary to mesenteric plexiform neurofibroma [8]. Intraoperative findings in our patient of serosal neurofibromatous lesions on the stomach and small bowel, in conjunction with congenital adhesions, suggest NF1 may have played a pathophysiological role.

### Surgical decision-making

Management of TCV is surgical. Simple detorsion alone carries recurrence rates of up to 70% and is generally not recommended [9]. Segmental resection may suffice if the bowel is viable and no redundancy is present, but subtotal colectomy is often preferred in cases of ischemia, megacolon, or extensive redundancy, as in our patient [10, 11]. Emma *et al*. [12] emphasized that in the presence of megacolon, subtotal colectomy reduces the risk of recurrence compared with segmental resection.

In elderly patients, surgical planning must balance physiological reserve with recurrence risk. Subtotal colectomy may carry greater operative stress but provides a more definitive solution. In our case, the extent of ischemia, dilatation, and mesenteric abnormalities justified a subtotal colectomy over segmental resection.

### Primary anastomosis versus stoma

The decision between primary anastomosis and stoma formation is nuanced. Primary anastomosis is feasible when the patient is stable, bowel ends are viable, and contamination is minimal. However, elderly patients and those with ischemic or contaminated fields face a significant risk of anastomotic leak. In our patient, an initial ileosigmoid anastomosis was attempted but subsequently leaked, necessitating re-laparotomy and ileostomy formation. This highlights the importance of tailoring surgical strategy not only to intraoperative findings but also to patient comorbidity and postoperative resilience.

### Histopathology

Histopathology confirmed transmural ischemia with associated serositis and inflammatory exudate. Reactive lymph nodes were present but no malignancy. Importantly, benign neurofibromatous lesions were identified, which strengthens the hypothesis of NF1 contributing to volvulus pathogenesis. The combination of histological ischemia and neurofibromatous changes reinforces the complexity of bowel involvement in NF1.

### Clinical implications

This case adds to the sparse literature linking NF1 with volvulus. Surgeons should maintain heightened suspicion for NF1-related gastrointestinal pathology in patients with unexplained obstruction. Furthermore, in elderly patients presenting with TCV, subtotal colectomy with a low threshold for stoma formation may represent the safest long-term strategy.

Clinical presentation is often nonspecific, with symptoms such as abdominal pain, constipation, and vomiting. Unlike sigmoid volvulus, abdominal distension may be less pronounced, which can delay diagnosis [7].

Radiology plays a crucial role; plain abdominal radiographs may show nonspecific distension but rarely establish the diagnosis. A CT scan is the gold standard [8], with the whirl sign, transition point, and proximal dilatation being diagnostic. CT also assesses complications such as ischemia and perforation, which carry high mortality [9].

Management is surgical; simple detorsion has a recurrence rate of 70%, so colectomy is the recommended treatment. Subtotal colectomy is preferred when bowel ischemia or redundancy is present. Mortality remains high (up to 30%) in delayed cases with gangrene [7, 10].

Our case highlights the importance of early radiological suspicion, especially in elderly patients with bowel obstruction and typical findings. Prompt CT diagnosis allowed timely surgical intervention, which was lifesaving [11].

## Conclusion

TCV, although rare, should be considered in the differential diagnosis of large bowel obstruction. CT imaging is crucial for preoperative diagnosis and assessing complications. Definitive management is surgical, with colectomy preferred over simple detorsion due to recurrence risk. Early recognition and intervention are key to reducing morbidity and mortality.

## References

[1] Kapadia M . Volvulus of the small bowel and colon. Clin Colon Rectal Surg 2016;30:040–5. 10.1055/s-0036-1593428 PMC5179272 28144211

[2] Gingold D, Murrell Z. Management of colonic volvulus. Clin Colon Rectal Surg 2012;25:236–44. 10.1055/s-0032-1329535 24294126 PMC3577612

[3] Al-Doud MA, Al-Omari MA, Dboush HG, et al. Large bowel obstruction as a consequence of transverse colon volvulus: a case report. Int J Surg Case Rep 2020;76:534–8. 10.1016/j.ijscr.2020.10.070 33207426 PMC7599370

[4] Lyons D . Sigmoid volvulus: a case series, review of the literature and current treatment. Am J Biomed Sci Res 2019;6:352–7. 10.34297/AJBSR.2019.06.001059

[5] Consorti ET, Liu TH. Diagnosis and treatment of caecal volvulus. Postgrad Med J 2005;81:772–6. 10.1136/pgmj.2005.035311 16344301 PMC1743408

[6] Zinkin LD, Katz LD, Rosin JD. Volvulus of the transverse colon. Dis Colon Rectum 1979;22:492–6. 10.1007/BF02586939 527437

[7] Ören D, Atamanalp SS, Aydinli B, et al. An algorithm for the management of sigmoid colon volvulus and the safety of primary resection: experience with 827 cases. Dis Colon Rectum 2007;50:489–97. 10.1007/s10350-006-0821-x 17205203

[8] Peterson CM, Anderson JS, Hara AK, et al. Volvulus of the gastrointestinal tract: appearances at multimodality imaging. Radiographics 2009;29:1281–93. 10.1148/rg.295095011 19755596

[9] Newton N, Reines H. Transverse colon volvulus: case reports and review. Am J Roentgenol 1977;128:69–72. 10.2214/ajr.128.1.69 401592

[10] Dholoo F, Shabana A, See A, et al. A rare cause of intestinal obstruction in late pregnancy: case report. Int J Surg Case Rep 2021;80:105391. 10.1016/j.ijscr.2020.11.141 33431333 PMC7982489

[11] Niksch J, Thurley N, Boutall A. Transverse colon volvulus—a case report and literature review. S Afr J Surg 2023;61:237–9. 10.36303/SAJS.4069 38450699

[12] Rosin E, Walshaw R, Mehlhaff C, et al. Subtotal colectomy for treatment of chronic constipation associated with idiopathic megacolon in cats: 38 cases (1979-1985). J Am Vet Med Assoc 1988;193:850–3. 3192467

